# The Common Collagen of Alport Syndrome and Arthritis: A Case Report and Review of Pathophysiology

**DOI:** 10.1155/crin/9705418

**Published:** 2026-07-01

**Authors:** Craig A. Mendonca, Lalarukh Haider, Anusha Attre, Eric M. Mortensen, Narinder Maheshwari

**Affiliations:** ^1^ Department of Medicine, University of Connecticut Health Center, 263 Farmington Ave, Farmington, Connecticut, 06030, USA, uchc.edu; ^2^ Department of Nephrology, University of Connecticut Health Center, 263 Farmington Ave, Farmington, Connecticut, 06030, USA, uchc.edu; ^3^ School of Medicine, University of Connecticut, 200 Academic Way, Farmington, Connecticut, 06032, USA, uconn.edu

**Keywords:** Alport syndrome, arthritis, case report, collagen, hematuria

## Abstract

**Aim:**

Through the lens of a clinical case, we explore the overlap in pathophysiology between Alport syndrome and seronegative inflammatory arthritis, focusing on the collagen structural changes that can occur in Alport syndrome.

**Results:**

Genetic variant information was obtained for a patient diagnosed with Alport syndrome. Few cases of Alport syndrome associated with inflammatory arthritis have been reported in the literature so far. The genes encoding collagen fibrils affected in Alport syndrome are also expressed in hyaline cartilage. The inflammatory response triggered by these misfolded, truncated, or missing proteins may contribute to inflammatory pathways present in arthritis through compromised collagen structures.

**Clinical Takeaway:**

Evaluation of hematuria and concomitant inflammatory arthritis should include consideration of hereditary connective tissue disorders, such as Alport syndrome.

## 1. Introduction

Alport syndrome is a systemic genetic disorder caused by a pathogenic variant in collagen‐producing genes, *COL4A3*, *COL4A4*, or *COL4A5* [1]. Because different types of collagen are expressed widely across various organs for structural and functional support, pathogenic variants can lead to diverse clinical presentations and disease associations. Most notably, Alport syndrome is linked to nephritis, chronic kidney disease (CKD), sensorineural deafness, retinopathy, and leiomyomatosis [1], depending on where the variant collagen genes are expressed. The joint space is another potential site where these genes can be expressed, producing proteins essential for movement. Changes in these proteins or their levels of expression could contribute to inflammatory pathways present in the development of arthritis.

Few cases have been reported involving patients with both arthritis and Alport syndrome. In one case [2], a 44‐year‐old man was diagnosed with RF and ACPA‐positive rheumatoid arthritis after workup for unexplained microhematuria. Electron microscopy revealed a thin GBM (130–200 nm) with segmental fusion of epithelial foot processes, and genetic analysis identified a *COL4A5* variant consistent with Alport syndrome. Another report [3] describes a 7‐year‐old boy with juvenile idiopathic arthritis, who also had persistent microscopic hematuria. Subsequent genetic testing revealed a *COL4A4* variant consistent with Alport syndrome. Rheumatoid arthritis and other forms of arthritis can have renal manifestations. Additionally, treatment for these conditions involves medications that can have nephrotoxic side effects, which may complicate the clinical picture. Therefore, alternative causes of hematuria must be carefully considered in patients with inflammatory arthritis.

## 2. Case Presentation

### 2.1. Initial Presentation

A 54‐year‐old woman with no known medical history presented to her primary care physician complaining of foot and hip pain. Workup showed normocytic anemia, and urinalysis revealed a large amount of hemoglobin and more than 50 RBCs per high‐power field. She was referred to urology, where cystoscopy showed inflammatory changes at the bladder neck but no evidence of cancer on biopsy. A vaginal lesion was identified as a benign fibroepithelial polyp. She also saw a GI specialist for chronic gastrointestinal bleeding and was diagnosed with Barrett’s esophagus and *H. pylori* infection. No polyps were found during colonoscopy. Despite treatment with iron, a PPI, and a combination of bismuth, tetracycline, and metronidazole, her anemia persisted.

Her initial lab work for her joint pain evaluation showed a weak positive ANA at 1:80 with a homogenous pattern, an ESR of 69 mm/hr, C3 level of 171 g/L, and a total complement of 168 units/mL. However, tests for rheumatoid factor, dsDNA, CCP, CRP, lactate dehydrogenase, C4, hepatitis B panel, TSH, and HLA‐B27 were negative.

### 2.2. Diagnosis and Genetics

Based on her elevated ESR, ANA, and negative antibody and biomarker panel, she was diagnosed with undifferentiated/seronegative inflammatory arthritis. Further classification of the seronegative inflammatory arthritis did not support seronegative rheumatoid, psoriatic, or gouty arthritis given normal bone radiography, whole body bone scan, and lack of dermal findings. Disease‐modifying antirheumatic drug (DMARD) therapy was postponed until further evaluation of her anemia could be completed. Since no source of microscopic hematuria was identified, she was referred to nephrology. Urine albumin‐to‐creatinine ratio was 42 mg/g, and urine protein‐to‐creatinine ratio was undetectable, so genetic testing was chosen over a kidney biopsy because of mild proteinuria. The Natera Kidney Gene Panel detected a heterozygous *COL4A4* pathogenic variant (c.1323_1340del p.Pro444_Leu449del). Follow‐up Invitae testing confirmed this pathogenic variant and found she is heterozygous for an additional variant of uncertain significance in *COL4A4* c.2045A > G (p.Asp682Gly). These results are consistent with autosomal dominant Alport syndrome.

**FIGURE 1:**
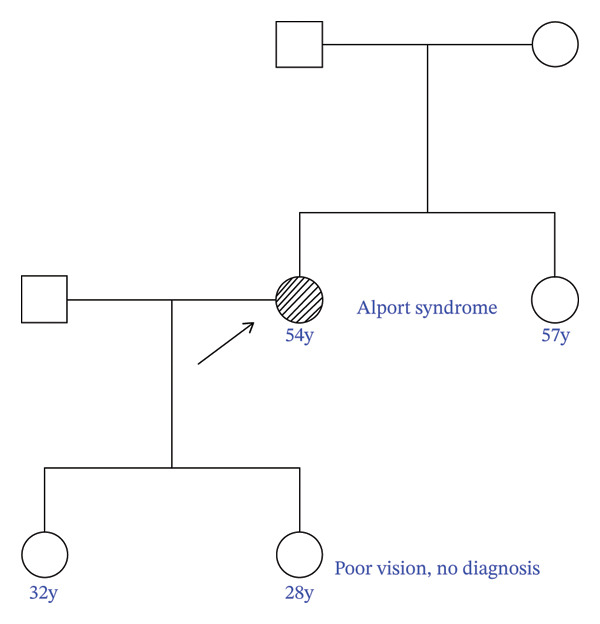
Pedigree diagram, without history of inflammatory conditions or Alport syndrome reported among available family members. The proband has a COL4A4 variant. Of note, the patient was separated from her biological parents at a young age.

The patient had been separated from her biological parents at a young age and was unaware of any significant medical conditions for them. She has a healthy sister and two daughters. One of her daughters has poor eyesight but no formal inflammatory syndrome diagnosis. Neither daughter has known hematuria or symptomatic anemia; however, the patient is not certain about the testing status of her family members (Figure 1). The patient was encouraged to contact her family members for urinalysis screening as hematuria is present in greater than 50% of patients who carry an autosomal dominant Alport syndrome variant [1]. There are no known inflammatory disorders that run in her family.

She had a 4‐year history of tinnitus and worsening subjective hearing difficulty but was found to have bilateral cerumen impaction. Removal of the cerumen by otolaryngology resolved her hearing issues, and subsequent formal audiometry was normal. An ophthalmologic evaluation is in progress.

### 2.3. Treatment and Outcome

She was started on DMARD therapy for seronegative arthritis: methotrexate 15 mg weekly, folic acid daily, and prednisone 10 mg daily. The daily prednisone was transitioned to Depo‐Medrol injections approximately every 6 months. This regimen provided relief from her seronegative inflammatory arthritis.

## 3. Discussion

The pathophysiology of both arthritis and basement membrane disease involves damage to collagen fibrils and networks. Notably, the two case reports from the literature review and the case presented in this article showed inflammatory arthritis and microhematuria occurring simultaneously [2, 3]. To evaluate the copresentation of these conditions, it is necessary to examine the gene expression, protein remodeling, and molecular structure of these collagen tissues.

### 3.1. Collagen Expression

The major collagens found in hyaline articular cartilage are Type II, IX, and XI [4]. However, minor collagen types also play vital roles in maintaining cartilage structure. Type IV collagen consists of *α*1 and *α*2 chains (*COL4A1* and *COL4A2,* respectively) which form a network. The relative mRNA expression of *COL4A3*, *COL4A4*, and *COL4A5* is very low in healthy articular cartilage [5]. These genes typically form the *α*3*α*4*α*5 isoform, which is commonly expressed in the basement membrane of the kidney, ear, and eye tissues affected in Alport syndrome [1]. Due to their low expression in mature articular cartilage [5], these genes were previously thought not to significantly influence joint function.

However, a recent study [6] has shown a temporal relationship between cartilage development and repair and the expression of different collagen isoforms. Type IV collagen is expressed early in the chondrocyte differentiation process. Therefore, genetic analysis of mature articular collagen may not fully reflect the impact of other isoforms. Fragments of Type IV collagen are released during remodeling and are present in both normal and osteoarthritic articular cartilage [7]. While this is not specific to inflammatory arthritis, the resulting chondrocyte remodeling process is shared.

### 3.2. Collagen Damage and Inflammation

Inflammation in the synovium has been shown to alter both collagen expression and degradation. In healthy synovial tissue, immunohistochemical staining revealed the predominance of *α*1*α*2 chains and the presence of *α*5 chains (expressed by *COL4A5*). Patients with rheumatoid arthritis exhibited markedly reduced expression of *α*1*α*2 but maintained *α*5 chain expression [8], indicating a potential reliance on other forms in inflammatory arthritis. qRT‐PCR of cultured synovial fibroblasts also demonstrated trace levels of *α*3 and *α*4 chains, suggesting the presence of the minor *α*3*α*4*α*5 isoform within normal synovial function. Interestingly, stimulating synovial fibroblasts with TNFα was associated with a more dramatic decrease in the dominant *α*1 and *α*2 chains compared to the *α*3, *α*4, and *α*5 chains [8]; however, statistical significance was not tested. These *α*3, *α*4, and *α*5 chains, which are affected in Alport syndrome, may influence joint structure or function in inflammatory arthritis, but further research is needed to reach definitive conclusions.

### 3.3. Collagen Destruction

Matrix metalloproteinases (MMPs) are involved in breaking down collagen chains. They are upregulated and released in both osteoarthritis and rheumatoid arthritis due to damage to chondrocytes and synovial cells [9]. Some COL4A protein variants have been linked to producing collagen variants that are more susceptible to MMP‐2, MMP‐3, and MMP‐9 [10]. Each of these MMP isoforms has different affinities for each collagen isoform. This increased susceptibility may lead to enhanced collagen breakdown and remodeling in patients with these variants. Overexpression of MMP‐13 has been shown to increase expression of Type IV collagen in chondrocytes [7], indicating a dynamic balance of collagen expression. Autoantibodies specific to denatured Type IV collagen fibrils have been found in patients with rheumatoid arthritis and juvenile idiopathic arthritis; however, antibodies against normal Type IV collagen fibrils were absent [11]. Therefore, an autoimmune response to misfolded collagen proteins could worsen an inflammatory arthritis.

## 4. Conclusion

In conclusion, we presented a case of seronegative inflammatory arthritis presenting with Alport syndrome and reviewed similar reports in the literature. Both inflammatory arthritis and Alport syndrome involve inflammation of collagen structures. Faulty or truncated collagen fibrils may play a role in the development of various forms of arthritis, beyond those typically linked to collagen in Alport syndrome. While this case and those previously reported are observational, the shared Type IV collagen network expression, activity, and degradation provide a plausible underlying mechanism. Further research in this area will be required to establish causality. Nevertheless, unexplained hematuria in the context of inflammatory arthritis should alert clinicians to the possibility of disorders involving connective tissue abnormalities.

## Author Contributions

Craig A. Mendonca and Narinder Maheshwari conceptualized the project, Craig A. Mendonca drafted the manuscript and figures, and Craig A. Mendonca, Lalarukh Haider, Anusha Attre, and Narinder Maheshwari critically revised and approved the manuscript.

## Funding

This study was not supported by any sponsor or funder. This work was performed at the University of Connecticut Health Center.

## Ethics Statement

Written informed consent was obtained from the patient for publication of this case report. No personally identifiable or protected health information is included in this report.

## Conflicts of Interest

Craig A. Mendonca is listed as an inventor on patent US20230089490A1, in collaboration with the University of Massachusetts Amherst. This patent covers gene therapy techniques applicable to various genes, including those mentioned in this report. All other authors report no financial conflicts of interest.

## Data Availability

Data sharing is not applicable to this article as no datasets were generated or analyzed during the current study.
